# Pharmacologic and non-pharmacologic strategies to prevent intracranial pressure surges during endotracheal suctioning in acute brain injury: a narrative review

**DOI:** 10.1186/s12871-026-03615-3

**Published:** 2026-01-14

**Authors:** Sagar Jolly, Shashank Paliwal, Rashida Lokhandwala, Aditya Gadepalli, Kiran Jangra, Navneh Samagh, Abhijit Vijay Lele, Rafi Avitsian

**Affiliations:** 1 Department of Anesthesiology, New York Medical College, Metropolitan Hospital Center, 1901 1 st Ave, New York, NY 10029 USA; 2 Department of Anaesthesiology, Critical Care and Pain Medicine, All India Institute of Medical Sciences, Bathinda, Punjab India; 3 Department of Anaesthetics, University Hospitals Coventry and Warwickshire, Coventry, UK; 4 Anaesthetics and Intensive Care, Royal Free London NHS Foundation Trust, London, UK; 5 Department of Anaesthesia and Intensive Care, Postgraduate Institute of Medical Education and Research, Chandigarh, India; 6 Departments of Anesthesiology and Pain Medicine. Neurology and Neurological Surgery, Harborview Medical Center, University of Washington, Seattle, USA; 7 Department of Anesthesiology, Cleveland Clinic, Cleveland, OH USA

**Keywords:** Mechanical ventilation, Endotracheal suctioning, Intracranial pressure, Cerebral blood flow, Cerebral perfusion pressure, Traumatic brain injury, Secondary brain injury

## Abstract

Endotracheal suctioning remains a critical intervention in the respiratory management of mechanically ventilated patients with brain injury. However, it carries the risk of triggering abrupt intracranial pressure elevations, potentially exacerbating secondary brain injury. This narrative review combines current evidence on pharmacologic and non-pharmacologic strategies to mitigate ICP fluctuations during ETS in neurosurgical patients. The findings support the need for an integrated approach involving analgo-sedation, controlled ventilation, and targeted drug selection to minimize ICP surges and improve outcomes. While each strategy offers distinct benefits, its application must be adapted to the patient’s physiological profile and clinical context, and regional resources. The review also highlights the need for continued research into effective dosing, administration techniques, and the integration of real-time monitoring technologies to enhance procedural safety in this high-risk population.

**Supplementary Information:**

The online version contains supplementary material available at 10.1186/s12871-026-03615-3.

## Introduction

 Endotracheal suctioning (ETS) is routinely performed in mechanically ventilated patients, including those with acute brain injury (ABI) to maintain airway patency and prevent pulmonary complications such as atelectasis and ventilator-associated pneumonia, especially in those with impaired or absent cough reflex. However, ETS can induce abrupt elevations in intracranial pressure (ICP), transiently reduce cerebral perfusion pressure (CPP), and raise concern for exacerbating secondary brain injury [1, 2]. These effects are driven by a physiological cascade triggered by suctioning, including cough reflex activation, sympathetic discharge, and changes in intrathoracic pressure, notably in patients with impaired cerebral autoregulation [3, 4]. In routine practice, ETS is performed multiple times, so these physiologic perturbations may occur frequently over the course of an ICU stay.

ETS is often performed repeatedly over days of ventilation, so transient ICP surges may recur many times during an ICU stay. In patients with severe TBI, the ICP “dose” (the pressure–time integral above a 20 mm Hg threshold over the first 24–72 h) has shown a progressively stronger association with 30-day mortality and 3- and 6-month functional outcomes than merely the presence or number of 5-minute ICP elevations [5]. Subsequent burden-mapping analyses have suggested that sustained ICP in the 15–20 mmHg range is linked to worse outcomes, whereas higher values may be tolerated when very short-lived, depending on CPP and autoregulatory reserve [6, 7]. Thus, frequent ETS-related peaks may cumulatively increase ICP dose, supporting strategies that blunt the height and duration of suction-induced surges.

In the 1970 s, Williams was among the earliest to systematically quantify that cerebrospinal fluid (CSF) pressure elevations upon coughing can significantly raise ICP, a finding later reinforced in studies linking ETS-induced cough to ICP spikes in patients with severe head injury [2, 3]. Multiple studies have demonstrated that ETS can transiently raise ICP by 10 to 20 mmHg, particularly in the absence of adequate sedation or neuromuscular blockade [2, 4, 8, 9].

Despite its widespread use, there is still no standardized strategy for optimal sedation, suctioning technique, and timing in relation to ICP trends for ETS [4]. Given that secondary brain injury strongly influences long-term outcomes after brain trauma, establishing evidence-based strategies for ETS is essential to minimize iatrogenic harm. This review brings together and evaluates pharmacologic and non-pharmacologic strategies including current evidence on sedatives, local anesthetics, neuromuscular blockers, ventilation strategies, and suctioning techniques, and aligns these interventions with neurophysiological mechanisms to guide safer ETS practices for patients at elevated risk of secondary brain injury.

## Methodology

Three authors (R.L., S.P., S.J.) independently performed a literature search across PubMed, Embase, Scopus, and Web of Science. Although no medical librarian was formally involved, the authors devised the search strategy to cover relevant literature. Search strategy used keywords and Boolean operators covering terms related to intracranial pressure, intracranial hypertension, brain injury, and endotracheal suctioning, as outlined in Supplementary Table 1. We included all eligible studies published from 1990 to 2025, reflecting the limited literature on ETS-related ICP surges, and used 1990 as a pragmatic starting point aligned with modern neurocritical care practices.

**Fig. 1:**
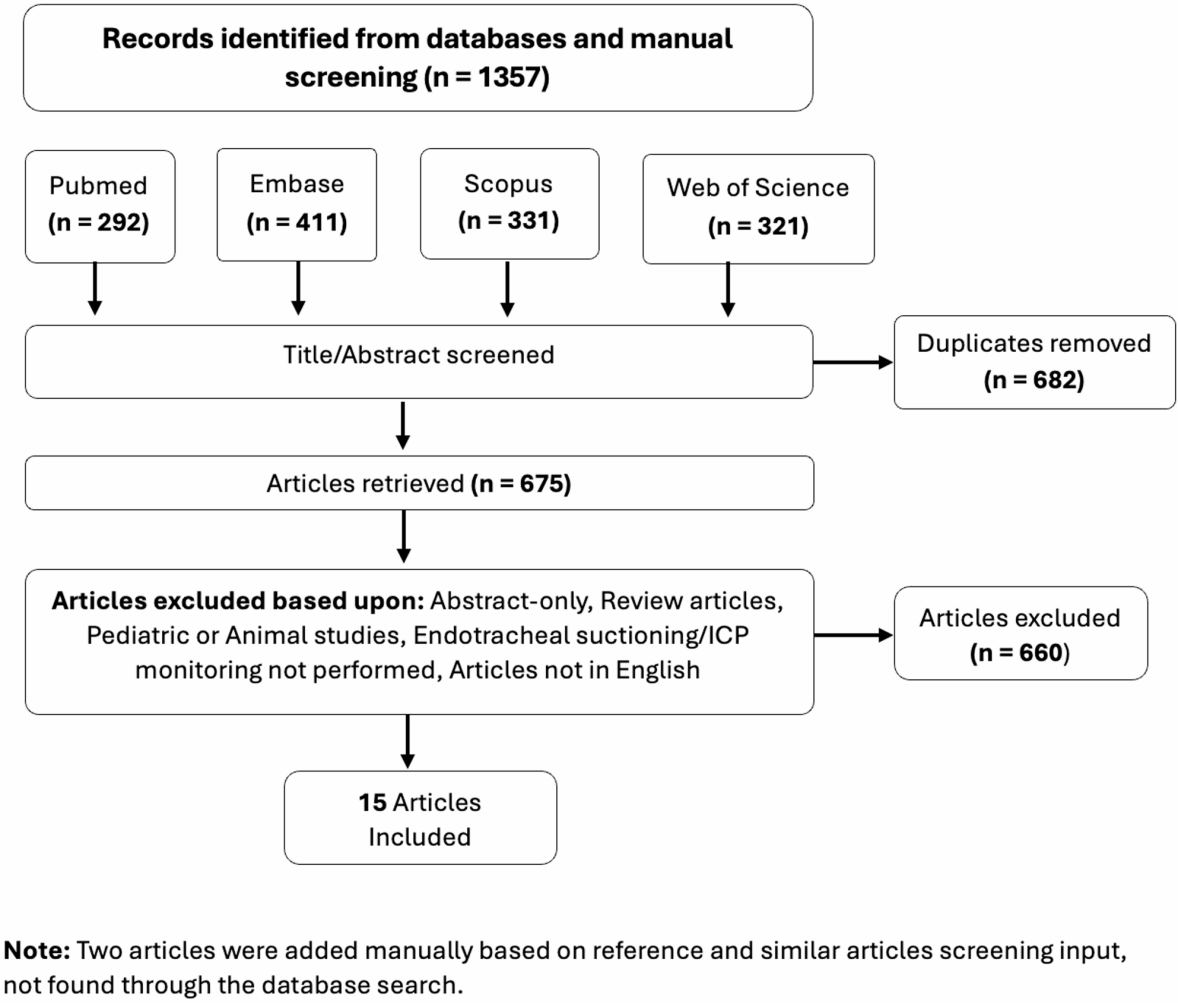
Search strategy

**Fig. 2:**
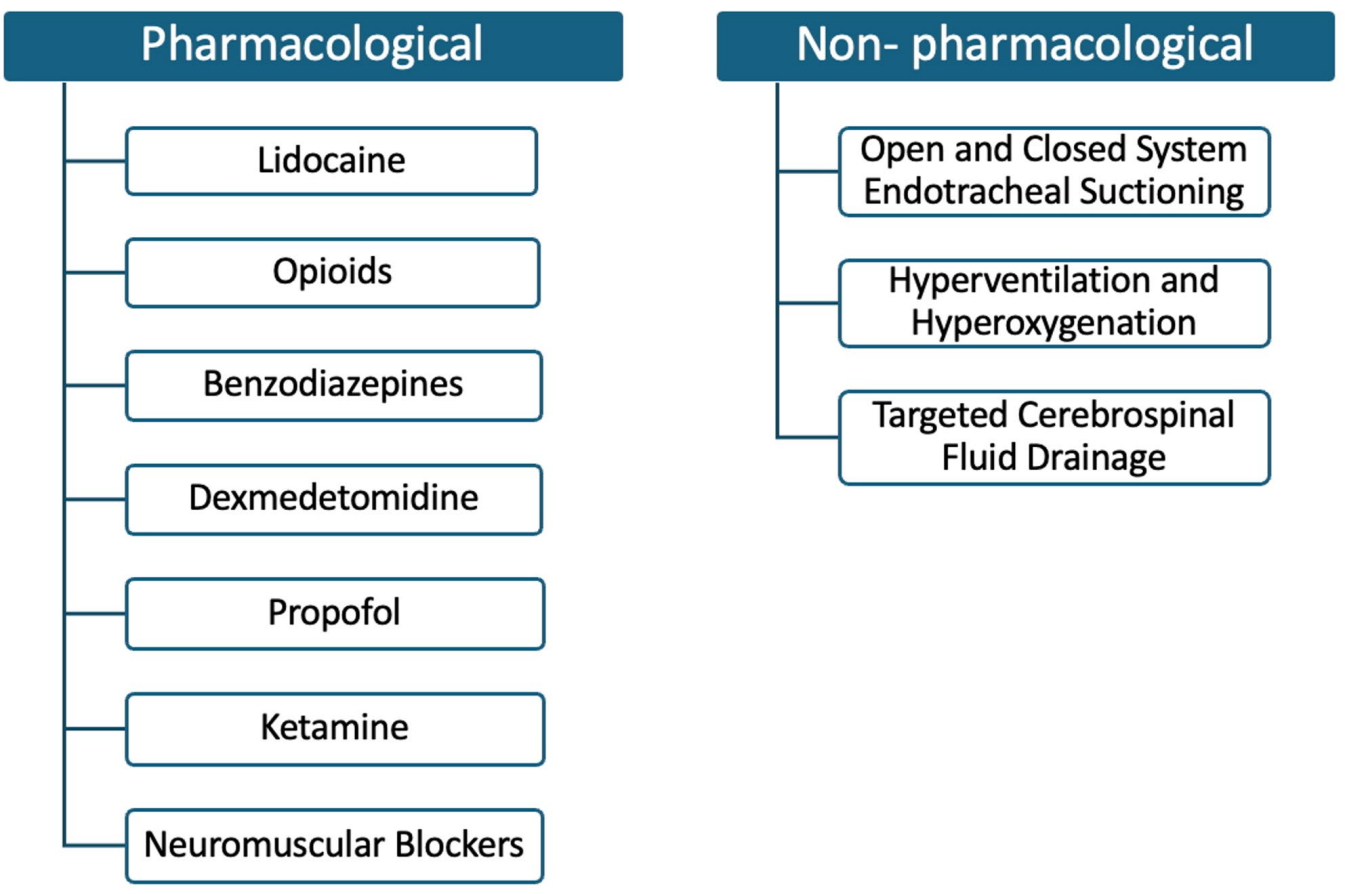
Overview of therapeutic modalities for intracranial pressure control during endotracheal suctioning

Two authors (S.P. and R.L.) independently reviewed all titles, abstracts, and full-text articles to determine eligibility. Disagreements were resolved through discussion and consensus involving the third author (S.J.). Review articles, pediatric or animal studies, non-English publications, and studies without ICP monitoring during ETS were excluded (Fig. 1). After screening and assessment, 15 articles were included for this narrative review (Table 1). Selected studies were on mechanically ventilated adults with ABI where ETS was the direct target of the intervention, evaluating the effectiveness of both pharmacological and non-pharmacological strategies (Fig. 2) for the prevention of ICP elevations during ETS.

**Table 1:** Studies investigating the effect of different modalities to prevent ICP changes during endotracheal suctioning

Study (Author and Year)	Methodology	Population and Sample Size	Baseline Sedation	Study Variables	Hemodynamic Effects of Intervention	Outcomes of Tested Interventions	Cough Reflex	Additional Findings
Bilotta et al. [10] (2008)	Prospective, observer-blinded, crossover clinical trial	Adult Severe TBI patients; *n* = 41	Propofol infusion at 3–5 mg/kg/hr	ET Lidocaine 2 mg/kg, titrated ± 0.5 mg/kg, to prevent ICP rise ≥ 20 mmHg	No hypotension observed	ET Lidocaine ED_50_ = 1.7 ± 0.3 mg/kg effectively prevented post-suction ICP↑ and CPP ↓ in 50% responders	Not Suppressed	ET lidocaine instillation may induce coughing, increasing ICP in patients; therefore, ED95 is preferred for reliable ICP suppression.
Rodrigues et al. [11] (2013)	Prospective, Randomized Controlled Study	Adult Severe TBI patients; *n* = 60 (3 groups, 20 each)	Baseline sedation and analgesia present in all groups	Group 1: IV Lidocaine 1.5 mg/kg Group 2: ET Lidocaine 1 mg/kg Group 3: No Lidocaine	No hypotension observed	IV (*P* = 0.56) and ET Lidocaine (*P* = 0.06) prevented ICP ↑, CPP stable Control group: ICP ↑ (*P* = 0.0002), CPP ↓	Not Suppressed	ET Lidocaine safety comparable to IV lidocaine if plasma levels stay non-toxic.
Mathieu et al. [12] (2013)	Prospective, blinded, crossover clinical trial	Adult patients with Severe SAH, ICH, TBI; *n* = 15	Midazolam + Sufentanil infusion	6 mL of 0.9% aerosolized NaCl vs. 6 mL of 2% aerosolized lidocaine (2 mg/kg)	No hypotension observed	ETS increased ICP by 6 ± 2 mmHg (*P* < 0.01) and reduced CPP by 2 ± 2 mmHg (*P* < 0.01) after NaCl; prevented with Lidocaine (ΔICP: 1 ± 1 mmHg; ΔCPP: −1 ± 1 mmHg, *P* < 0.05)	Not Suppressed	Limit aerosolized lidocaine to acute phase to prevent cumulative toxicity risk.
Singh et al. [13] (2018)	Prospective, Randomized study	Adult Severe TBI patients; *n* = 60 (2 groups, 30 each)	Fentanyl 1 µg/kg/hr + Midazolam 0.1 mg/kg/hr	IV Dexmedetomidine 0.5 µg/kg vs. IV Lidocaine 2 mg/kg, both over 10 min	Dexmedetomidine: ↓ MAP and CPP (*P* < 0.001), MAP remained >65 mmHg; Lidocaine: no significant MAP, CPP changes	Both drugs blunted ICP ↑ (*P* > 0.05), MAP ↑, and HR ↑ after ETS; Lidocaine preferred due to stable CPP and MAP	Not Suppressed	Study included Normal baseline ICP patients; no sedation score was documented; results may not generalize to patients with raised ICP.
Hanowell et al. [14] (1993)	Prospective, blinded clinical trial	Adult Severe TBI patients; *n* = 7	None	Alfentanil 15–30 µg/kg vs. Saline, administered over 1 min; ETS performed 1 min later	Alfentanil ↓ MAP and CPP	Alfentanil did not prevent ICP ↑; CPP significantly ↓ with Alfentanil (*P* < 0.05)	Not Suppressed	Alfentanil-associated CPP reduction requires further studies.
Leone et al. [15] (2004)	Prospective observational (crossover) study	Adult Severe TBI with haemodynamic stability (ICP < 25 mmHg for 4 h and CPP > 70 mmHg for ≥ 24 h); *n* = 20	None	Dose 1: 1 µg/kg bolus + 0.25 µg/kg/min × 30 min; Dose 2: 2 µg/kg bolus + 0.5 µg/kg/min × 30 min; Dose 3: 4 µg/kg bolus + 1 µg/kg/min × 30 min; each dose separated by 60-min washout. ETS performed 20 min post-bolus	MAP ↓, CPP ↓ in all groups; vasopressor use: Dose 1 = 12/20, Dose 2 = 15/20, Dose 3 = 19/20 (*P* < 0.01 Dose 2, *P* < 0.001 Dose 3)	ICP ↑ during ETS despite Remifentanil (*P* < 0.05 all doses); coughing suppressed dose-dependently; EC50 for cough suppression: 17.1 ± 6.2 ng/mL	Dose-dependent suppression; absent in 75% at highest dose	Higher doses modestly attenuated ICP rise but required vasopressors for CPP; not recommended as sole ETS agent in TBI.
Robin et al. [16] (2017)	Prospective, Randomized Controlled Pilot Study	Adult Male Severe TBI patients; *n* = 20	Midazolam 0.2 mg/kg/hr + Fentanyl 0.002 mg/kg/hr	Midazolam group (2 mg IV bolus 1 min before ETS) vs. Control (no bolus)	Not reported	Mean ICP rise: Control = 24.1 ± 11.1 mmHg, Midazolam = 18.25 ± 9.29 mmHg (*P* < 0.05); Total ICP variation from baseline: Control = 28.4 ± 0.68 mmHg, Midazolam = 20.8 ± 0.48 mmHg (*P* < 0.05)	Not suppressed	Time to ICP normalization post-ETS: Control = 15 min, Midazolam = 10 min (*P* < 0.05).
Wu et al. [17] (2020)	Prospective Randomized Controlled Trial	Adult patients after craniocerebral surgery for cerebrovascular disease, brain tumors, or severe brain injury, with ICP monitoring and initial ICP ≤ 25 mmHg; *n* = 208	None	Experimental group: 104 patients, IV propofol (10 ml with 1 ml 2% lidocaine) 0.5–1 mg/kg, pre-ETS; Control group: 104 patients, ETS alone	Systolic BP ↑ after ETS; Propofol attenuated rise (*P* = 0.008)	Propofol group had lower mean ICP rise (15.6 ± 12.3 vs. 18.2 ± 9.0 mmHg; *P* < 0.0001) despite high variability; choking cough response (*P* < 0.01) and ICP fluctuation ↓ with Propofol (*P* < 0.0001)	Reduced significantly with Propofol (*P* < 0.01)	Pre-ETS propofol reduced choking cough and ICP fluctuations, improving 6-month neurosurgical prognosis (Higher Glasgow Outcome Scale; *P* = 0.037).
Gemma et al. [1] (2002)	Prospective, non-randomized observational study	Adult Severe TBI patients; *n* = 17	None	Propofol 2–6 mg/kg/hr or Diazepam 0.1–1 mg/kg/hr during ETS, with no NMBs	Propofol or Diazepam sedation maintained MAP ↑ (+ 7 ± 12 mmHg) and CPP ↑ (+ 6 ± 14 mmHg) post-ETS; inadequate sedation led to greater CPP ↓ (Δ −7 ± 15 mmHg; *P* = 0.003)	Propofol or Diazepam sedation limited ICP ↑ (Δ + 2 ± 6 mmHg); inadequate sedation caused greater ICP ↑ (Δ + 13 ± 6 mmHg; *P* < 0.0001) and SjO₂ ↓ (Δ −3 ± 5%; *P* < 0.0001)	Suppressed in adequately sedated patients	With inadequate sedation, Post-ETS ICP rise lacked compensatory CBF increase and drop in SjO_2_ drop may indicate stress-induced elevated cerebral metabolism.
Caricato et al. [18] (2013)	Prospective observational study (2-phase approach)	Adult Severe TBI within 72 h; *n* = 21	Propofol 3–5 mg/kg/hr + Remifentanil 0.05–2 µg/kg/hr (Analgosedation); Ramsay score 5–6	Control: Analgosedation alone; Intervention: Analgosedation + Racemic Ketamine 100 µg/kg/min for 10 min pre-ETS	MAP ↑ in Control phase (89.0 ± 11.6 → 96.4 ± 13.1 mmHg; *P* < 0.001); MAP stable with Ketamine	ICP ↑ post-ETS in both phases; attenuated with Ketamine (Control: 11.0 ± 6.7 → 18.5 ± 8.9 mmHg; *P* < 0.001; Ketamine: 11.0 ± 6.4 → 15.1 ± 9.4 mmHg; *P* < 0.05); CPP unchanged	Significantly suppressed with Ketamine (*P* < 0.0001)	Ketamine did not effectively control ICP rise after ETS at administered doses.
De Cerqueira Neto et al. [19] (2013)	Prospective, non-randomized clinical trial	Adult Severe TBI within 48 h; *n* = 20	Propofol + Pancuronium, Ramsay Scale 6	VBC and IEFM for 5 min per hemithorax, followed by 5-ml saline instillation, three hyperinflation breaths with hyperoxygenation, then ETS performed	MAP ↑ after ETS (Δ + 6.4 mmHg; *P* = 0.011); MAP stable after VBC/IEFM	VBC/IEFM did not alter MAP, ICP, or CPP; ETS caused ICP ↑ (Δ + 6.7 mmHg; *P* < 0.001); CPP stable due to MAP ↑	Suppressed with sedation and paralysis	VBC and IEFM did not affect ICP or CPP; ETS altered cerebral hemodynamics despite sedation and paralysis, though CPP remained stable due to coexistent MAP increase.
Werba et al. [20] (1993)	Prospective observational clinical Trial with crossover design	Adult Severe TBI and Spontaneous SAH; *n* = 18	Midazolam 5 mg/hr + Sufentanil 1 µg/kg/hr	Propofol 0.5 mg/kg bolus vs. NMBs (Group A - Vecuronium 0.12 mg/kg or Group B - Atracurium 0.4 mg/kg) pre-ETS	Not reported	↑ ICP and ↓ CPP after Propofol bolus (*P* < 0.05); NMBs prevented ICP ↑ and CPP ↓; post-ETS ICP and CPP unchanged with Vecuronium (ICP 20 ± 8 to 20 ± 8 mmHg; CPP 65 ± 13 to 65 ± 13 mmHg) and Atracurium (ICP 19 ± 7 to 19 ± 7 mmHg; CPP 62 ± 12 to 65 ± 11 mmHg)	Moderate-to-severe coughing in all patients post-ETS with Propofol alone; suppressed after NMB bolus	TOF absence does not ensure diaphragm paralysis; PTC 5 with Vecuronium or 1 with Atracurium was required to suppress diaphragmatic movement during ETS.
Kerr et al. [21] (1998)	Secondary analysis of Prospective quasi-experimental design	Adult Severe TBI; *n* = 71 (3 groups)	None	Group 1: No drugs; Group 2: Opioids only (morphine/fentanyl); Group 3: NMBs (vecuronium) + opioids	Not reported	ICP ↑ during ETS in No-drug and Opioid-only groups (*P* < 0.05); NMBs + opioids attenuated ICP rise vs. other groups (*P* < 0.05)	Not reported	Opioids alone did not reduce ICP rise, subjects were already on opioids at baseline. NMB-induced diaphragm/intercostal paralysis likely responsible for ICP attenuation during ETS.
Uğraş and Aksoy et al. [22] (2012)	Prospective crossover, Single-Blind clinical trial	Adult neurosurgical ICU patients; *n* = 32	None	16 patients underwent CSS followed by OSS, and the next 16 vice versa, with suction techniques alternating post-baseline normalization	Both CSS and OSS significantly increased MAP (*P* < 0.01); OSS caused greater MAP rise (*P* < 0.001)	ICP ↑ with both techniques (*P* < 0.01); CSS limited rise during, post-ETS, and at 5, 15 min vs. OSS (*P* < 0.01); no CPP difference (*P* > 0.05)	Not suppressed	Both CSS and OSS ↑ ICP, MAP, CPP, HR; PaO₂ higher with CSS (*P* = 0.038); PaCO₂ unchanged; CSS considered safer option.
Kerr et al. [23] (1997)	Prospective, quasi-experimental, crossover (repeated measures)	Adult Severe TBI patients; *n* = 66	None	Control: 4 breaths/20 s (12/min) pre-ETS and post-ETS. Protocol 1: 8 breaths/40 s (12/min) pre-ETS and post-ETS. Protocol 2: 4 breaths/8 s (30/min) pre-ETS and post-ETS.	MAP ↑ in all groups; greater fluctuations with 30-breath protocol (*P* = 0.02); CPP significantly ↑ with 30-breath protocol (*P* = 0.004)	ICP rise significantly reduced with 30-breath hyperventilation vs. 4-breath control during suction phases (*P* = 0.001); no significant reduction with 8-breath protocol (*P* = 0.18)	Not suppressed	HV may induce an inverse steal phenomenon in ischemic regions.

*TBI* Traumatic Brain Injury, *SAH* Subarachnoid Hemorrhage,* VBC* Vibrocompression, *IEFM* Increased Expiratory Flow Manual, *TOF* Train-of-four, *ETS* Endotracheal Suctioning, _*ED95*_ 95% Effective Dose, _*SjO2*_ Jugular venous oxygen saturation,* OSS* Open System Suctioning,* CSS* Closed System Suctioning

### Studies investigating pharmacological strategies

#### Lidocaine

Lidocaine, a widely used local anesthetic, is used to attenuate ICP elevations associated with ETS in sedated neurosurgical patients [10–13]. IV lidocaine, with a rapid onset (45–90 s) and short duration (10–20 min), is often given before intubation to blunt airway reflexes [24]. It has minimal impact on blood pressure and does not interfere with neurological exams, making it suitable for managing transient noxious stimuli like ETS in brain-injured patients [25]. From a pharmacokinetics perspective, with a terminal half-life of 1.5 to 2 h, IV lidocaine could be administered as boluses every 3 to 4 h in patients with normal hepatic function; however, further studies are needed to validate concerns for cumulative toxicity [26]. Both endotracheal (ET) and intravenous (IV) routes have demonstrated efficacy, although they act through distinct mechanisms. ET lidocaine primarily suppresses the cough reflex at the airway mucosa, whereas IV lidocaine exerts systemic effects by inducing cerebral vasoconstriction [27]. Although a substantial difference has not been described, a downside of ET administration of lidocaine is that the amount reaching the trachea is unreliable and can provoke coughing; also the endotracheal secretions may dilute the lidocaine, decreasing its effectiveness [28]. It may also inhibit the cough reflex via sodium channel blockade, inhibition of pulmonary vagal afferents, and relaxation of airway smooth muscle, seen in preclinical studies [29, 30]. However, its central effects remain debated. Notably, Poulton et al. reported unclear evidence on lidocaine’s suppressive effect on brainstem function for cough suppression [31]. Ultimately, the choice between the two routes of administration depends on the patient’s specific clinical condition and the desired balance between local and systemic effects.

In a dose-escalation crossover study by Bilotta et al., ET lidocaine (1.7 ± 0.3 mg/kg) effectively prevented ICP surges > 20 mmHg in 50% of severe traumatic brain injury (TBI) patients with preserved CPP without adverse systemic effects [10]. However, the authors noted that ET instillation can paradoxically provoke coughing if directly administered. To minimize this, they recommended warming the solution to body temperature and instilling it slowly (1 mL/sec) via a fine catheter passed along the ET tube, avoiding direct mucosal contact at the tip. This technique seems most applicable to planned suctioning and may be impractical during urgent or unanticipated ETS. In a related study, Rodrigues et al. found that neither ET (1 mg/kg) nor IV (1.5 mg/kg) lidocaine significantly reduced mean ICP following suctioning, yet both effectively prevented elevations above 20 mmHg. Bilotta et al., in contrast, evaluated titrated-dosing response and the proportion of patients whose ICP remained below 20 mmHg, while Rodrigues reported group-level mean changes with fixed-dosing, which are two fundamentally different endpoints that complicate direct comparison and reflect distinct clinical priorities [11]. This suggests that the clinical benefit lies in avoiding harmful ICP spikes rather than altering mean values.

Singh et al. demonstrated that IV lidocaine (2 mg/kg) effectively blunted ICP and cardiovascular responses, with greater hemodynamic stability than dexmedetomidine [13]. Although lidocaine is widely available in hospital formularies for airway anesthesia in procedures such as bronchoscopy and intubation, its aerosolized/nebulized form use remains underreported and underappreciated in ICU literature. In a randomized crossover study, Mathieu et al. found that aerosolized lidocaine (2 mg/kg) significantly attenuated ICP elevation following ETS, limiting the peak ICP increase to 1 ± 1 mmHg versus 6 ± 2 mmHg with saline, without affecting systemic or cerebral hemodynamics [12].

These findings suggest that lidocaine is a potential adjunct for attenuating ICP elevations during suctioning in sedated intubated patients with ABI. It can be administered endotracheally, intravenously, or via aerosol. Across studies, effective lidocaine doses range from 1.0 to 2.0 mg/kg, varying by administration route: 1.0–2.0 mg/kg for ET instillation (ED₅₀ ≈ 1.7 mg/kg), 1.5–2.0 mg/kg IV, and 2.0 mg/kg aerosolized. These dosages are practical for clinical use, though further research is needed to clarify long-term neuroprotective effects.

#### Opioids

Studies suggest that opioids such as fentanyl, sufentanil, and alfentanil can raise ICP due to vasodilatory effects following systemic hypotension in patients with severe TBI [32, 33]. Despite this, analgesia and sedation are commonly utilized to reduce ICP and prevent secondary brain injury. Alfentanil and Remifentanil were investigated for their rapid onset and controlled duration, essential in managing ICP during ETS.

##### Alfentanil

Alfentanil, a short-acting opioid with a rapid onset (1–2 min) and duration of action of 30–60 min, is a practical choice for procedural analgesia in critically ill patients. However, its use during ETS in patients with TBI requires caution due to its impact on systemic hemodynamics and cerebral perfusion.

Hanowell et al. administered alfentanil at 15 µg/kg and 30 µg/kg three minutes before ETS in sedated, ventilated patients with severe TBI. Both doses failed to prevent a rise in ICP, which peaked to ~ 22 mmHg in the 15 µg/kg group and ~ 34 mmHg in the 30 µg/kg group, compared to ~ 27 mmHg in the saline control. These changes were accompanied by a reduction in mean arterial pressure (MAP) and a resulting decline in CPP reaching a nadir near 49 mmHg in both dosing groups, approaching the threshold for compromised cerebral oxygen delivery [14]. Although not clearly specified, the reduced MAP and increased ICP suggested that autoregulatory vasodilation from systemic hypotension may have raised cerebral blood volume and ICP, rather than a direct vasodilatory effect of alfentanil [34].

Albanese et al. administered a 100 µg/kg bolus of alfentanil followed by continuous infusion (0.75 µg/kg/min) in patients with severe head injury. Despite sedation, paralysis, and mild hypocapnia, ICP increased transiently by 5.5 ± 1.0 mmHg, peaking 3 min post-bolus and returning to baseline within 15 min. This was accompanied by MAP reduction of 26 ± 2 mmHg and a CPP decline of 34 ± 3 mmHg [32]. The authors attributed the ICP rise to autoregulatory vasodilation secondary to hypotension. While no ischemic events occurred, the magnitude of CPP decline highlighted the potential risk of bolus alfentanil in patients with compromised intracranial compliance.

Taken together, these small studies data suggest that large bolus doses of alfentanil given immediately before ETS may cause clinically significant reductions in MAP and CPP in severe TBI. Given these hemodynamic concerns, including autoregulatory cerebral vasodilation and the likelihood of impaired autoregulation, we suggest exercising caution with high bolus dosing during ETS, instead using lower bolus doses (≤ 10 µg/kg) or carefully titrated infusions with a sedation regimen, with close CPP monitoring [34–36].

##### Remifentanil

Remifentanil is an ultra-short-acting µ-opioid agonist, with rapid onset (1–3 min.) and terminal half-life (10–20 min.), allowing for predictable recovery, valuable in neurocritical care, where neurological reassessment is frequent. At low-to-moderate infusion rates (0.05 to 0.25 µg/kg/min), remifentanil maintains stable ICP and CPP in ventilated brain-injured patients, provided MAP is preserved [37, 38].

Leone et al. studied 20 severe TBI patients, comparing three remifentanil bolus doses (1, 2, and 4 µg/kg) followed by infusions (0.25, 0.5, and 1.0 µg/kg/min), using a crossover design with suctioning 20 min into each infusion. Complete cough reflex suppression occurred only at high concentrations, with an EC50 of 17.1 ± 6.2 ng/mL; 3–5 times higher than standard general anesthesia concentrations. This benefit was accompanied by a slight increase in ICP to 22 ± 9, 19 ± 7, and 19 ± 8 mmHg with each dose, respectively. CPP dropped to 59–63 mmHg due to MAP reduction without impairing cerebral autoregulation with preserved VmMCA (middle cerebral artery mean flow velocity), and most patients (60%, 75%, and 95% respectively) needed vasopressor support with each dose [15].

Although remifentanil offers pharmacokinetic advantages, its high cost limits prolonged ICU sedation. It effectively suppresses cough during ETS in TBI patients, demonstrating a dose-response effect and maintaining cerebral autoregulation. Infusion rates of 0.25 µg/kg/min or higher may increase ICP and lower CPP, often leading to vasopressor use. Given its cost, fentanyl boluses or higher infusion rates are often used as a less expensive alternative. In patients with ABI and reduced intracranial compliance, remifentanil should be titrated carefully with close hemodynamic monitoring. More prospective ETS studies are needed to clarify its dose-response and safety in brain-injured patients.

### Benzodiazepines

#### Midazolam

Midazolam, a short-acting, water-soluble benzodiazepine, acts via GABA-A (gamma-aminobutyric acid) receptor potentiation, resulting in sedative, anxiolytic, muscle relaxant, and anticonvulsant properties [39]. It is widely used as a sedative because it reduces the cerebral metabolic rate of oxygen (CMRO2) and cerebral blood flow (CBF), and moderately lowers ICP while causing minimal hemodynamic instability compared to agents like propofol [40, 41]. A randomized study by Robin et al. showed that administering 2 mg midazolam one minute before ETS in severe TBI patients receiving continuous midazolam–fentanyl sedation significantly reduced the mean rise in ICP after suctioning (18.25 ± 9.29 vs. 24.1 ± 11.1 mmHg) and the mean variation of ICP from baseline during suctioning (20.8 ± 0.48 vs. 28.4 ± 0.68 mmHg), and shortened the time for ICP to return to baseline (10 vs. 15 min) compared with no bolus [16]. Though both groups experienced some elevation from baseline, indicating midazolam alone may not fully prevent ICP increases during ETS.

Midazolam may not fully prevent suction-related ICP surges, but as a sedation adjunct, it reduces their intensity and duration without significant effects on hemodynamics in severe TBI patients. Although it appears to offer a favorable risk-benefit profile, further studies are needed on its dosing and its role alongside other ICP-targeted interventions during ETS. However, due to its confounding effect on neurological assessment, it is rarely used for ICU sedation except in indications like status epilepticus.

#### Remimazolam

Remimazolam is an ultra-short-acting benzodiazepine introduced for procedural sedation and general anesthesia in 2020, with ongoing research into its use in intensive care. It is rapidly metabolized by tissue esterases, preventing accumulation even in patients with hepatic or renal impairment, and provides stable hemodynamics with minimal respiratory depression [42].

There are no studies yet on remimazolam’s effects in brain-injured patients or during ETS. Early clinical data indicate it may reduce ICP increases in situations like steep Trendelenburg positioning and permissive hypercapnia. In a randomized trial of laparoscopic gynecologic surgery in Trendelenburg position with pneumoperitoneum, both remimazolam and sevoflurane groups demonstrated an increase in ONSD (Optic Nerve Sheath Diameter) over time; however, the time-dependent increase was significant only with sevoflurane, and the percentage rise at 30 min was slightly smaller with remimazolam (6.9% vs. 8.4%) [43]. However, the ONSD changes were not attributed solely to the anaesthetic, given the concurrent effects of positioning, pneumoperitoneum, and hypercapnia. A plausible explanation is that volatile agents such as sevoflurane may augment CO₂-mediated cerebral vasodilation and increase cerebral blood volume, whereas benzodiazepines primarily reduce cerebral metabolic rate with a proportionate decrease in CBF. Whether these physiologic differences result in clinically meaningful changes in ICP, particularly among brain-injured patients undergoing ETS, remains uncertain and warrants further targeted investigation.

### Dexmedetomidine

Dexmedetomidine (DEX), a selective α2-agonist, provides sedation without significant respiratory depression in neurocritical care, supporting neurological assessment and ventilator synchrony in TBI patients [44]. This is especially important in TBI, where impaired autoregulation and perfusion-metabolism mismatch can exacerbate secondary brain injury.

In healthy individuals, DEX reduces cerebral metabolic rate (CMR) and CBF in a dose-dependent manner, with jugular venous oxygen saturation (SjO₂) remaining stable, indicating preserved oxygen extraction and CMR–CBF coupling despite vasoconstriction [45]. In severe TBI patients, DEX lowers sedative and opioid needs without impairing neurological function or causing significant hemodynamic instability [44]. Khallaf et al. randomized 60 agitated TBI patients with ICP monitoring to DEX, propofol, or their combination for sedation titrated to maintain ICP < 20 mmHg and CPP > 60 mmHg across the three groups, and observed no major differences in frequency of ICP > 20 mmHg or CPP < 50 mmHg over 48 h [46][]. In a separate retrospective before–after cohort study of 23 neurosurgical ICU patients with refractory intracranial hypertension, initiation of adjunctive DEX was associated with fewer mannitol rescue doses but no differences in hypertonic bolus use, external ventricular drain output, ICP crises, or episodes of hypotension, bradycardia, or low CPP events. However, the retrospective design and small sample size substantially limited causal inferences that DEX itself improved ICP control [47].

DEX has been shown to lower CBF and CMR, indicating maintained cerebral oxygenation even with reduced perfusion [48]. However, Singh et al. found that DEX (0.5 µg/kg), compared to lidocaine (2 mg/kg), both reduced the increase in ICP during suctioning, but DEX caused a greater drop in MAP and CPP, which may risk cerebral perfusion, especially in patients with impaired intracranial compliance [13]. Although DEX has previously shown maintained oxygenation despite reduced CBF under hypercarbia, these results may not apply to real-world suctioning conditions under normocarbia [45].

DEX may benefit ETS by improving ventilator synchrony, reducing sedative requirements, and lowering cerebral metabolism. However, it can also cause bradycardia and hypotension, with risk of reduced CPP, particularly in those with impaired autoregulation. A rapid IV bolus may trigger transient hypertension via peripheral α2B adrenoceptor stimulation on vascular smooth muscle, potentially worsening ICP with disrupted autoregulation. Current evidence suggests its use during ETS should be guided by close hemodynamic monitoring to avoid compromising CBF until safety and efficacy are more clearly established.

### Propofol

Propofol, a rapid-onset sedative with neuroprotective and anticonvulsant properties, is widely used in neurocritical care due to its favorable cerebral hemodynamic profile. It lowers CBF, ICP, and CMRO₂, and in patients with intact cerebral pressure autoregulation, preserves autoregulatory function [49–51]. Its rapid onset (~ 30 s) and short context-sensitive half-time ranging from approximately 3 min after brief infusion to ~ 18 min following 12 h of continuous administration, makes it ideal for titratable sedation with predictable emergence for neurologic reassessment [52]. Additionally, it dilates bronchial smooth muscle and suppresses airway reflexes, helping prevent ICP spikes during airway manipulation. When combined with short-acting opioids such as remifentanil, propofol effectively suppresses airway reflexes during tracheal suctioning [53].

Wu et al. conducted a randomized controlled trial in which intravenous propofol (0.5–1 mg/kg mixed with 2% lidocaine) was given before ETS to postoperative brain-injured patients. The propofol group had significantly lower mean peak ICP during suctioning (15.57 ± 12.31 mmHg vs. 18.24 ± 8.99 mmHg), reduced coughing, and better long-term outcomes (51.5% vs. 32.6%, Glasgow Outcome Scale 4–5 at 6 months) [17]. However, lidocaine administration introduced a confounding factor, and the large standard deviations indicated high individual variability, making it difficult to attribute effects solely to propofol.

A non-randomized study by Gemma et al. found that, during the first week of TBI, patients who coughed or moved due to inadequate sedation had significantly greater ICP surges (mean ΔICP 13 ± 6 mmHg) than well-sedated patients (2 ± 6 mmHg) [1]. Additionally, CPP and SjO₂ dropped in inadequately sedated patients but improved in the well-sedated cohort. Therefore, maintaining adequate sedation depth is important to blunt physiological responses to ETS and maintain cerebral oxygenation.

Propofol bolus, especially in hypovolemic patients, may cause systemic hypotension and reflexly increase ICP via cerebral vasodilation. Clinical evidence supports the use of bolus doses of 0.5–1 mg/kg, with or without an infusion at 3–5 mg/kg/h, titrated according to sedation targets. When combined with lidocaine and short-acting opioids, propofol appears effective in minimizing airway reflexes and mitigating ETS-induced ICP elevations. However, differences in study design, sedation depth, and co-administered agents limit direct comparisons. Further controlled trials are needed to refine sedation protocols during ETS and assess long-term neurologic outcomes [10, 17].

### Ketamine

Ketamine, a noncompetitive NMDA (N-methyl-D-aspartate) receptor antagonist, has re-emerged as a valuable sedative in neurocritical care due to its dissociative anesthetic properties and minimal hemodynamic compromise. Unlike other sedatives, ketamine preserves MAP and respiratory drive, making it particularly suitable for patients with impaired autoregulation or hypotension, where maintaining CPP is critical. These, along with bronchodilation and neuroprotective effects, led to renewed interest in its use for patients with elevated ICP [54–57].

Ketamine’s pharmacologic effects are stereoselective; the S (+) enantiomer (esketamine) binds more strongly to NMDA receptors, producing sedation at lower doses compared to the R(-) isomer. In a porcine model of raised ICP, racemic ketamine (10 mg/kg IV) reduced ICP by 10.8%, whereas the isolated enantiomers had no such effect. All three ketamine formulations, racemic, S (+), and R(–), produced a biphasic CBF response, with an initial 25–29% decrease followed by a transient rebound of 7–15%, along with dose-dependent MAP reductions. Although only racemic ketamine significantly lowered ICP (10.8%), its supratherapeutic dosing (10 mg/kg) and associated hypotension limits clinical applicability [58].

Level II clinical evidence indicates that ketamine does not increase ICP when used under controlled ventilation and co-administered with GABAergic agents [59]. In children with refractory intracranial hypertension, bolus doses of 1–1.5 mg/kg lowered ICP by 30% and improved CPP without systemic hypotension [60]. Recent studies indicate that ketamine is safe in hypotensive trauma, where its MAP-preserving properties may protect CPP and reduce secondary brain injury [55]. Evidence (Oxford level 2b, Grade C) suggests that ketamine does not increase and may reduce ICP in sedated, normocapnic, mechanically ventilated patients while supporting CPP [56].

In a crossover study on severe TBI patients sedated with propofol and remifentanil, ETS significantly raised ICP, MAP, SjO₂, and mean VmMCA, without affecting CPP. Adding a 10-minute racemic ketamine infusion (100 µg/kg/min) before repeat ETS reduced the cough reflex and maintained MAP, CPP, VmMCA, and SjO₂; however, ICP still rose modestly from baseline (11.0 ± 6.4 to 15.1 ± 9.4 mmHg), indicating partial suppression of suction-induced ICP response with possible dosing or timing limitations [18].

Ketamine has shown hemodynamic stability and a neutral-to-favorable ICP profile in brain-injured patients on controlled ventilation. Paradoxically, its secretagogue properties may increase airway secretions and the frequency of ETS. Although it may reduce ETS-related ICP surges, uncertainties remain about dosing strategies, timing, and enantiomer selection. Without prospective neurological outcome trials, ketamine is still a valuable sedation option for brain-injured patients, particularly when hypotension, opioid tolerance, or respiratory suppression limit the use of alternative agents.

### Neuromuscular blockade

Non-depolarizing neuromuscular blockers (NMBs) cause skeletal muscle paralysis by competitively inhibiting post-synaptic nicotinic receptors at the neuromuscular junction [61]. In neurocritical care, NMBs are used with sedation and analgesia to facilitate mechanical ventilation, improve oxygenation, and reduce patient–ventilator desynchrony [62]. In brain-injured patients with elevated ICP, neuromuscular blockade can help prevent an abrupt increase in pressure by reducing coughing during ETS [20].

De Cerqueira Neto et al. observed that in mechanically ventilated severe TBI patients sedated with propofol and paralyzed with pancuronium, respiratory physiotherapy did not significantly affect MAP, ICP, or CPP. However, suctioning transiently increased ICP (19.7 ± 8.2 to 26.4 ± 12.8 mmHg) and MAP (94.3 ± 18.4 to 100.7 ± 22.2 mmHg), normalizing within 10 min, with CPP remaining stable. Despite deep sedation (Ramsay score 6) and neuromuscular blockade with cough reflex suppressed, these findings suggested that suctioning can provoke ICP elevations, likely from carinal stimulation and sympathetic activation in the absence of analgesia [19]. Therefore, authors suggested performing ETS combining sedation, analgesia, and neuromuscular paralysis to attenuate ICP fluctuations and maintain cerebral hemodynamics.

Werba et al. analyzed the depth of neuromuscular blockade to prevent ETS-induced ICP elevations by suppression of diaphragmatic movement in sedated TBI and subarachnoid hemorrhage (SAH) patients under controlled ventilation (PaCO₂ 30 ± 2 mmHg). A 0.5 mg/kg propofol bolus did not suppress coughing or prevent ICP and CPP changes during suctioning. When vecuronium or atracurium was administered twice the ED95 dose, and deep blockade was confirmed with a TOF (TOF count zero, post-tetanic count < 5), suctioning did not alter ICP or CPP [20]. These results indicate that deep neuromuscular blockade verified by quantitative monitoring can suppress airway reflexes, limit ICP surges during suctioning, and maintain CPP in neurocritical care.

Kerr et al. studied 71 severe TBI adults who underwent two sequential suctioning trials after single hyperoxygenation phase. Each trial involved hyperinflation followed by ETS. Both hyperinflation and ETS increased ICP, but opioid and neuromuscular blocker use reduced ICP spikes during ETS compared to non-paralyzed controls. However, hyperinflation raised ICP regardless of paralysis from raised intrathoracic pressure and reduced venous return. The authors proposed that ETS-induced ICP elevations resulted from sympathetic activation, increased intrathoracic pressure, and reduced cerebral venous outflow [21]. These findings supported selective use of neuromuscular blockade during suctioning to blunt ICP surges, noting that hyperinflation-induced elevations are less responsive to paralysis due to distinct physiological mechanisms.

Evidence supports the use of short-term neuromuscular blockade to facilitate ventilation in patients with ABI [62]. However, paralysis increases secretion burden and the need for frequent ETS, which can raise ICP during suctioning. Agents like atracurium may cause hypotension via histamine release, affecting CPP. Prolonged NMB use increases risks of ICU-acquired weakness, delayed weaning and neurological evaluation, especially in patients with impaired consciousness or seizure risk [63]. When used selectively and for limited periods, NMBs help suppress cough-induced ICP spikes and improve ventilator synchrony; their administration should be targeted and closely monitored to balance benefits against potential risks and complications.

### Studies investigating non-pharmacological strategies

#### Effect of open and closed endotracheal suctioning on icp

Endotracheal suctioning using either open (OSS) or closed system suctioning (CSS) clears secretions, maintains airway and tube patency, and reduces pulmonary infection risk. OSS requires ventilator disconnection, while CSS maintains circuit continuity, preserves lung volume, and reduces hypoxemia compared to OSS [64].

A crossover, single-blind trial in 32 neurosurgical ICU patients showed that OSS caused higher ICP spikes than CSS immediately and up to 15 min after suctioning (OSS: 21.03 ± 8.81 mmHg vs. CSS: 16.28 ± 8.00 mmHg). Both methods increased MAP, CPP, and HR in the absence of sedation, but PaO₂ was consistently higher with CSS, and PaCO₂ levels were similar [22]. These findings suggest that CSS better limits ICP increases and preserves oxygenation during ETS.

A systematic review by Galbiati et al. analyzed 14 studies of critically ill neurosurgical patients, found that both OSS and CSS increase ICP; however, OSS more often raises ICP above 20 mmHg [65]. Overall, while OSS is linked to higher ICP during suctioning, CSS provides greater hemodynamic stability and oxygenation, making it a preferable choice for neurocritical patients. Transitioning from OSS to CSS requires coordinated efforts with respiratory therapists regarding equipment, training, and suctioning protocols.

#### Controlled hyperventilation and hyperoxygenation prior to ets

Hyperventilation (HV) lowers PaCO2, causing cerebral vasoconstriction and CBF, which decreases ICP in TBI patients. For each mmHg decrease in PaCO2, CBF drops by about 2–4% [66]. Though HV can help manage ICP temporarily, there is a risk of ischemia if PaCO2 goes below 26 mmHg [67]. The cerebrovascular response to CO₂ is mainly driven by perivascular pH changes that affect the vascular tone of cerebral arterioles [68]. With prolonged HV, compensatory CSF buffering can normalize perivascular pH and lessen vasoconstriction, even with ongoing hypocapnia [69]. Thus, HV’s duration, depth, and timing should be carefully managed, especially in patients with impaired cerebral autoregulation [67, 70].

HV and hyperoxia both cause cerebral vasoconstriction, but affect oxygen delivery and tissue metabolism differently [71, 72]. Research on targeted hyperoxia after ABI has shown mixed neurological results, highlighting uncertainty about its benefits [73–75]. In a quasi-experimental, crossover study of adults with severe TBI, Kerr et al. compared a standard 4-breath control protocol with two interventions: 8-breath and 30-breath sequences of hyperventilation and hyperoxygenation (HV/HO) administered immediately before ETS [23]. The 30-breath HV/HO approach significantly lowered ETS-induced ICP spikes while maintaining CPP through raised MAP and reduced ICP. Importantly, no rebound ICP occurred, likely because the brief hypocapnia prevented CSF pH compensation seen with prolonged HV.

Evidence supports that short-to-intermediate hyperoxygenation (≤ 3 h) is physiologically tolerable for brain-injured patients, not adversely affecting ICP, CBF, and brain tissue oxygenation (PbtO₂) [76, 77]. While concerns remain about potential vasoconstriction and reduced perfusion in ischemic areas, worsening secondary brain injury. Thus, carefully titrating short-term HO/HV before ETS can help control procedure-related ICP spikes without harming cerebral physiology, provided it is tailored to patient-specific and closely monitored.

#### Targeted cerebrospinal fluid drainage

CSF drainage effectively lowers ICP by reducing intracranial volume, allowing continuous monitoring and therapeutic diversion in ABI patients. In a prospective study, Candanedo et al. demonstrated that draining 4 mL of CSF lowered ICP by 7 mmHg (26 → 19 mmHg) in TBI patients, while non-TBI patients needed 7 mL to achieve a 6 mmHg reduction (19 → 13 mmHg), indicating reduced compliance in TBI [78]. Similarly, in a dose-response study, Kerr et al. showed a rapid, volume-dependent ICP reduction: 1, 2, and 3 mL drains led to 3.0, 3.4, and 4.5 mmHg drops, respectively, within a minute [79]. Kerr et al.‘s ETS study used CSF drainage for ICP elevations above 20 mmHg, but its individual effect on ETS-related ICP spikes was not separately assessed [80]. These studies suggest targeted, low-volume CSF drainage may help reduce transient ICP surges before ETS in brain-injured patients, though its specific effect during ETS still requires further evaluation.

CSF drainage is a standard Tier One intervention for elevated ICP, but its ability to reduce suction-related ICP spikes lacks strong direct evidence and needs more well-designed prospective studies.

### Unaddressed gaps in icp management protocols for ets: insights from trials and guidelines

Major randomized trials, including BEST TRIP, BOOST-II, and BOOST-3, have evaluated structured ICP management in severe TBI [81–83]. Pharmacologic strategies described across these trials include sedation, analgesia, hyperosmolar therapies (e.g., mannitol or hypertonic saline), neuromuscular blockade, and mild hyperventilation (PaCO₂ ~30–35 mmHg). The BEST TRIP trial protocol noted the potential use of IV or ET lidocaine to address respiratory agitation that could elevate ICP but did not specify measures for ICP surges during ETS. Non-pharmacological interventions included elevating the head of the bed (~ 30°), neutral head position, CSF drainage, and strict temperature management, maintaining normothermia and preventing hyperthermia. The protocol recommended frequent sterile tracheal suctioning as routine care to minimize pulmonary complications; it did not specifically address ICP control during suctioning episodes.

The benefit of ICP monitoring for improving clinical outcomes is debated. The BEST TRIP trial found no significant difference in functional or cognitive outcomes at six months when comparing ICP-guided treatment to a clinical examination and imaging-based protocol. BOOST-II showed that adding PbtO₂ monitoring to ICP-guided care reduced cerebral hypoxia and suggested trends toward lower mortality and improved outcomes, but lacked power for clinical efficacy. The BOOST-3 trial, adequately powered to detect a 10% difference in favorable outcomes, was designed to determine whether combined ICP and PbtO₂ monitoring improves functional outcomes; results are pending.

Current clinical guidelines lack specific recommendations for managing ETS-related ICP elevations. The 2017 Brain Trauma Foundation (BTF) Guidelines provide 28 evidence-based recommendations for ICP monitoring and treatment with modalities like CSF diversion, hyperosmolar therapy, and sedation, but did not address ETS-induced ICP elevations [67]. The ACS-TQIP Best Practice Guidelines emphasize the prevention of ventilator-associated pneumonia, early tracheostomy, and tiered neuroprotective measures, yet provide no specific guidance on ETS-specific strategies [84]. The 2019 SIBICC (Seattle International Severe Traumatic Brain Injury Consensus Conference) algorithm outlined a tiered protocol for intracranial hypertension, escalation from sedation, hyperosmolar therapy, CSF diversion, to decompressive craniectomy, but did not consider suctioning as a trigger or intervention point [85].

Together, these high-quality trials and guidelines provide a strong foundation for ICP control, but lack a targeted, evidence-based approach to mitigate suction-induced intracranial hypertension. Given the frequency of ETS in neurocritical care and its known physiological impact, this represents a critical and actionable knowledge gap, one that justifies further inquiry into real-world practices and protocol development, highlighting an evidence gap this review aims to bridge.

Various pharmacologic and non-pharmacologic strategies have been studied to reduce ETS-induced ICP responses, but the most effective approach remains unclear. Ideally, agents used before ETS should act quickly, last long enough, minimally impact ICP, MAP, and CPP, and not affect neurological assessments. It is uncertain whether increasing the dose of one sedative or combining two for an additive or synergistic effect is more effective. Combined use of short-acting opioids and sedatives may attenuate nociceptive and cough-mediated ICP surges, provided hemodynamic stability is maintained. While ET lidocaine can reduce airway reflexes to improve procedure tolerance, its slower onset, inconsistent absorption, and risk of airway irritation make it less reliable than IV administration in time-sensitive situations. Neuromuscular blockade facilitates controlled ventilation and reduces cough-induced ICP elevations, but its use should be time-limited due to concerns regarding ICU-acquired weakness and impaired neurological monitoring.

Most ETS-related ICP studies are small, single-center, and were conducted decades ago, predominantly in adults with severe TBI, with inclusion of SAH or mixed neurosurgical ICU populations. Sedation regimens, baseline ICP/CPP targets, ETS techniques (open vs. closed systems, hyperoxygenation, brief hyperventilation), and other co-interventions varied substantially between studies. Although ICP, CPP, and related neuromonitoring variables were typically primary outcomes around individual suctioning episodes, follow-up was limited to minutes to hours, and no studies have quantified cumulative ICP burden or the contribution of repeated suctioning episodes, nor reported long-term neurological outcomes. As a result, existing data does not allow a precise estimate of how much suction-related ICP “dose” contributes to secondary ischemic injury or functional outcome, and the most effective combination of measures to prepare patients for ETS remains uncertain.

Available evidence suggests that combining propofol with IV lidocaine and a short-acting NMB might offer the most consistent reduction of suction-related ICP surges, particularly in patients with impaired autoregulation or strong airway reflexes. When used in combination, these agents appear to provide synergistic control of pain, cough, and ventilation synchrony during ETS [10, 17, 20, 21]. Among non-pharmacologic interventions, CSS, brief hyperventilation, and oxygenation have shown benefit in minimizing ICP variability while preserving oxygenation. Adjusting these interventions to patient physiology, ICP trends, and procedural needs remains essential.

## Conclusion

Endotracheal suctioning in ventilated ABI patients can cause a sudden rise in ICP and may aggravate secondary brain injury. While pharmacologic and nonpharmacologic strategies can reduce these ICP surges, trials and guidelines do not define an ETS-specific best practice of an individualized, multimodal approach that prioritizes airway-reflex suppression and preservation of CPP.

Future studies should clarify optimal timing, dosage, drug combinations, and effects on long-term neurological outcomes. Improvements in real-time neuromonitoring, including continuous ICP and CPP tracking, can also guide safer suctioning and sedation protocols in neurocritical care.

## Supplementary Information

Supplementary Material 1: Table 1: Search Strategy: Studies published between 1990 and 2025; English language only.

## Data Availability

No datasets were generated or analysed during the current study.

## Abbreviations

ABI: Acute brain injury
TBI: traumatic brain injury
SAH: Subarachnoid hemorrhage
CBF: Cerebral blood flow
CMRO₂: Cerebral metabolic rate of oxygen
CPP: Cerebral perfusion pressure
SjO₂: Jugular venous oxygen saturation
PbtO₂: brain tissue oxygen partial pressure
VmMCA: middle cerebral artery mean flow velocity
ONSD: Optic Nerve Sheath Diameter
ETS: Endotracheal suctioning
OSS: Open system suctioning
CSS: Closed system suctioning
HO: Hyperoxygenation
HV: Hyperventilation
DEX: Dexmedetomidine
NMB: Non-depolarizing neuromuscular blocker
PTC: Post-tetanic count
TOF: Train-of-four
