# Supplementary material for: Pharmacologic and non-pharmacologic strategies to prevent intracranial pressure surges during endotracheal suctioning in acute brain injury: a narrative review
Source: BMC Anesthesiol. 2026 Jan 14;26:104. doi: 10.1186/s12871-026-03615-3 (PMC12888679; doi:10.1186/s12871-026-03615-3)
Supplement: Supplementary file 1 — Supplementary Material 1: Table 1: Search Strategy: Studies published between 1990 and 2025; English language only. [file 12871_2026_3615_MOESM1_ESM.docx]

**Table 1:** Search Strategy: Studies published between 1990 and 2025; English language only.

| Database | Search Query | Results |
| --- | --- | --- |
|  | | |
| PubMed | ("intracranial pressure"[Title/Abstract] OR "intracranial hypertension"[Title/Abstract] OR "brain injury"[Title/Abstract] OR "traumatic brain injury"[Title/Abstract] OR "TBI"[Title/Abstract] OR "severe head injury"[Title/Abstract] OR "head trauma"[Title/Abstract] OR "craniocerebral trauma"[Title/Abstract] OR "acute brain injury"[Title/Abstract])  AND  ("endotracheal suction"[Title/Abstract] OR "tracheal suction"[Title/Abstract] OR "airway suction"[Title/Abstract] OR "suctioning"[Title/Abstract] OR "closed suction system"[Title/Abstract] OR "open suction system"[Title/Abstract] OR "airway clearance"[Title/Abstract] OR "airway management"[Title/Abstract]) | **292** |
|  | | |
| Embase | ('intracranial pressure':ti,ab OR 'intracranial hypertension':ti,ab OR 'brain injury':ti,ab OR 'traumatic brain injury':ti,ab OR 'TBI':ti,ab OR 'severe head injury':ti,ab OR 'head trauma':ti,ab OR 'craniocerebral trauma':ti,ab OR 'acute brain injury':ti,ab)  AND  ('endotracheal suction':ti,ab OR 'tracheal suction':ti,ab OR 'airway suction':ti,ab OR 'suctioning':ti,ab OR 'closed suction system':ti,ab OR 'open suction system':ti,ab OR 'airway clearance':ti,ab OR 'airway management':ti,ab) | **411** |
|  | | |
| Scopus | (TITLE-ABS("intracranial pressure") OR TITLE-ABS("intracranial hypertension") OR TITLE-ABS("brain injury") OR TITLE-ABS("traumatic brain injury") OR TITLE-ABS("TBI") OR TITLE-ABS("severe head injury") OR TITLE-ABS("head trauma") OR TITLE-ABS("craniocerebral trauma") OR TITLE-ABS("acute brain injury"))  AND  (TITLE-ABS("endotracheal suction") OR TITLE-ABS("tracheal suction") OR TITLE-ABS("airway suction") OR TITLE-ABS("suctioning") OR TITLE-ABS("closed suction system") OR TITLE-ABS("open suction system") OR TITLE-ABS("airway clearance") OR TITLE-ABS("airway management")) | **331** |
|  |  |  |
| Web of Science | TS=("intracranial pressure" OR "intracranial hypertension" OR "brain injury" OR "traumatic brain injury" OR "TBI" OR "severe head injury" OR "head trauma" OR "craniocerebral trauma" OR "acute brain injury")  AND  TS=("endotracheal suction" OR "tracheal suction" OR "airway suction" OR "suctioning" OR "closed suction system" OR "open suction system" OR "airway clearance" OR "airway management") | **321** |

## **Additional Articles Identified**: In addition to the database search results, two articles (Rodrigues et al., 2013 [Ref. 8] and Robin et al., 2017 [Ref. 13]) were included based on manual screening of reference lists and similar articles, as they were not captured through the database queries.
